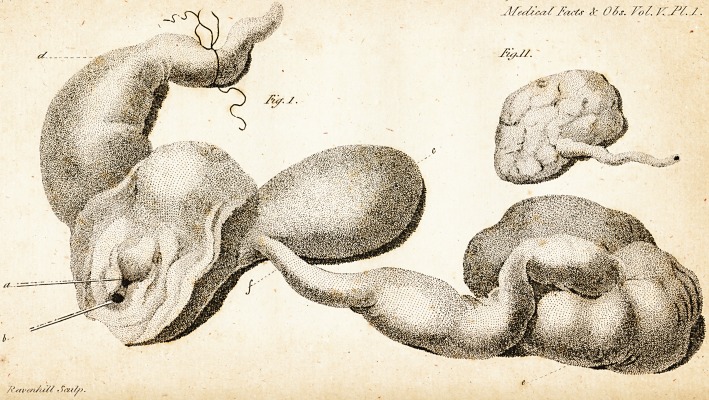# An Account of a Child Born without Organs of Generation

**Published:** 1794

**Authors:** Edward Ford

**Affiliations:** Surgeon to the Westminster General Dispensary.


### X. *An Account of a Child born without Organs of Generation. By Mr.* Edward Ford, *F. A. S. Surgeon to the Weſtminſter General Diſpenſary.*

IN December, 1792, I was deſired to ſee a child, born a few days before, with the anus imperforate in its uſual place, the fæces appearing to be diſcharged through the vagina. The external parts were remarkably ſmall in their conformation; the orifice of the meatus urinarius admitted a probe into the bladder; but on attempting to paſs a director, and afterwards a ſmall probe into the paſſage from which the fæces were diſcharged, I found it impracticable from the ſmallneſs of the aperture; and at the ſame time I diſcovered that there was no cavity ſimilar to the uſual one of the vagina.

It was propoſed a few days afterwards to form an artificial opening for the diſcharge of the fæces in the uſual ſituation of the anus, by introducing a probe into the preternatural opening, and puſhing it backwards, ſo as to project it below the os coccygis, and then to cut upon the inſtrument, in order to form, if poſſible, an outlet for the fæces, leſs liable to the inconveniences which muſt inevitably have reſulted from the unfortunate derangement of parts which at preſent ſubſiſted. A time was fixed for the operation, but the infant ſoon after becoming diſordered in its health, it was judged unneceſſary to attempt it.

The child died when it was three weeks old, and I had an opportunity of examining it after death, in the preſence of Dr. Jackſon, of Hanover Street, and Mr. Hunt, apothecary, of Swallow Street.

The firſt conſideration which preſented itſelf to my mind, was to perform ſuch an operation, on the dead body, as would have been thought requiſite to be done if the child had lived. In doing this, it was ſtill found impoſſible to paſs an inſtrument into the rectum from the external parts, till they were dilated downwards, in order to diſcover the orifice through which the fæces were diſcharged. This dilatation being effected, the aperture became viſible; and on preſſing the abdomen with my hand, the fæces were copiouſly diſcharged.

I then introduced a director into the rectum; and projecting it backwards, easily cut upon it, and, without much difficulty, formed an external communication with the gut in the part where the anus is usually formed. Had the child survived to have had this operation performed, it may be questionable, whether it would have fully answered the purpose of relieving the great inconvenience it laboured under. It might have been difficult to have procured the entire passage of the fæces through this artificial opening; and even had such an attempt succeeded, the want of a sphincter ani would have been a misfortune not likely to be supplied by art. Still, however, the disagreeable circumstance of living under so great a calamity as this child was born with, was sufficiently obvious to justify every attempt towards its relief.

On opening the cavity of the abdomen, and tracing the intestinal canal from the stomach, every part was found to be in a healthy state, and nothing preternatural occurred, till we came to the rectum, which was found to terminate close to the bladder, in the aperture before mentioned, just below the urinary passage.

The

Ravenhill Sculp.

The liver, gall bladder, ſpleen, and pancreas, were in a healthy and natural ſtate.

On removing the inteſtines, an unuſual prominence was obſerved on the left ſide, which proved to be the left kidney much enlarged; with its ureter dilated through its whole length (but principally at its origin) and terminating nearer to the neck of the bladder than uſual. The oppoſite kidney, on the right ſide, formed a ſtriking contraſt to the left; it was very ſmall and flat, and reſembled, in ſize and figure, a common bean; its ureter was about an inch in length, and had no connexion with the bladder.

The renal glands were wanting.

With regard to the contents of the pelvis, the bladder and rectum were as above deſcribed, contiguous to each other; and the termination of the rectum was cloſe to that of the urethra*: but

* In Fig. I. of the annexed engraving, (ſee Plate I.) the external parts are a little dilated, in order to ſhow the preternatural termination of the rectum a little below the meatus urinarius; *a* refers to a probe paſſed into the meatus urinarius; *b* to a probe paſſed into the anus; *c* to the fundus of the bladder; *d* to the rectum; *e* to the left kidney but upon the moſt accurate inſpection, both by Dr. Jackſon and ſeveral other gentlemen who have ſince viewed the preparation, it is evident that this child, which can ſcarcely be called a female, was born without either ovaria, uterus, or vagina.

---

XI. *Caſe of Apoplexy in a pregnant Woman; with Obſervations. By Mr.* Philip Williams, *Surgeon at Rugby in Warwickſhire. Communicated in a Letter to* John Clarke, M. D. *Teacher of Midwifery in London; and by him to Dr.* Simmons.

THE following caſe, which I offer to you, appears to me to deſerve attention, not only from the remarkable ſituation of the children, but alſo from the circumſtances attending kidney much enlarged; and *f* to the termination of the left ureter.

Fig. II. ſhows the right kidney, uncommonly ſmall.

## Figures and Tables

**Fig. I. Fig.II. f1:**